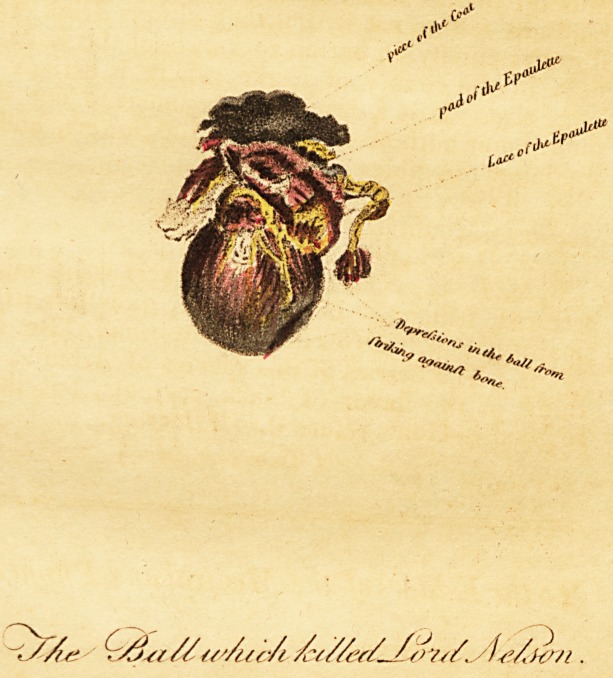# Mr. Beatty's Account of the Death of Lord Nelson

**Published:** 1806-01

**Authors:** W. Beatty

**Affiliations:** Surgeon. H. M. S. Victory


### *To the Editors of the Medical and Physical Journal.*

Gentlemen,

I Beg leave to transmit you a statement of the scite and nature of the wound which produced the death of the exceedingly lamented and late illustrious hero, Lord Nelson; and request you will please to insert it in the next number of the Medical and Physical Journal; enclosed is likewise a drawing of the fatal ball, with its appendages, which were carried before it through the whole course it described. *(Vide Engraving.)*

I am, &c.

W. BEATTY, Surgeon.

*H. M. S. Victory, Dec. 15, 1805.*

About

The Ball which killed Lord Nelson.

About the middle of the action with the combined fleets on the 21st of October last, the late illustrious commander in chief, Lord Nelson, was mortally wounded in the left breast by a musquet-ball, supposed to be fired from the mizen-top of La Redoubtable, French ship of the line, which the Victory fell on board of early in the battle; his Lordship was in the act of turning on the quarter deck, with his face towards the enemy, when he received his wound; he instantly fell, and was carried to the cockpit, where he lived about two hours.

On his being brought below, he complained of acute pain about the sixth or seventh dorsal vertebra, of privation of sense and motion of the body and inferior extremities; his respiration short and difficult, pulse weak, small, and irregular; he frequently declared his back was shot through; that he felt every instant a gush of blood within his breast; and that he had sensations which indicated to him the approach of death. In the course of an hour his pulse became indistinct, and was gradually lost in the arm; his extremities and forehead became soon afterwards cold. He retained his wonted energy of mind and exercise of his faculties until the latest moment of his existence; and when victory, as signal as decisive, was announced to him, he expressed his pious acknowledgments thereof and heart-felt satisfaction at the glorious event in the most emphatic langugage; he then delivered his last orders with his usual precision, and in a few minutes afterwards expired without a struggle.

*Course and Scite of the Ball, ascertained since Death.*

The ball struck the fore part of his Lordship's epaulette, and entered the left shoulder immediately before the processus acromion scapulæ, which it slightly fractured; it then descended obliquely into the thorax, fracturing the second and third ribs; and after penetrating the left lobe of the lungs, and dividing in its passage a large branch of the pulmonary artery, it entered the left side of the spine between the sixth and seventh dorsal vertebræ, fractured the left transverse process of the sixth vertebra, wounded the medulla spinalis, and fracturing the right transverse process of the seventh vertebra, it made its way from the right side of the spine, directing its course through the muscles of the back, and lodged therein, about two inches below the inferior angle of the right scapula.

On removing the ball, a portion of the gold lace and pad of the epaulette, together with a small piece of his Lordship's coat, were found firmly attached to it.

CRITICAL

## Figures and Tables

**Figure f1:**